# An agent-based approach to multi-scale neuronal network simulations using modified McCulloch-Pitts neurons

**DOI:** 10.1186/1471-2202-13-S1-P135

**Published:** 2012-07-16

**Authors:** Amanda L Hanes, Lee D Poeppelman, Jeffery M Gearhart

**Affiliations:** 1 Henry M. Jackson Foundation, USA; 2 711 HPW/RHDJ, 2729 R Street, Bldg 837, Wright-Patterson AFB, OH, 45433, USA

## 

A McCulloch-Pitts neuron is a simplified model of neuronal computation in which the neuron behaves as an adder; if the sum of the neuron's inputs passes a threshold, an action potential occurs. This type of neuron is invaluable for creating large network simulations because its simplicity allows a large number of neurons to be included, resulting in models with very simple neuronal mechanics but biologically accurate neuron counts and connectivity. The simulated neuronal activity in these models can approximate biological activity in the represented brain regions. By varying the firing threshold and including excitatory and inhibitory synapses (represented by positive and negative integer input to the neuron, respectively), a representation of different neuron types can be achieved, which adds to the accuracy of the resulting network activity. The drawback of this type of model is that the McCulloch-Pitts neuron is essentially a black box - it behaves approximately as a real neuron does, but contains none of the cellular basis for that activity and operates under the assumption that the neuron is exhibiting normal behavior. While it can accurately reproduce activity in the brain, there is no way to probe the relationship between that activity and the underlying neuronal mechanics upon which it is built, and no way to simulate the effects of injured or enhanced neurons.

We have developed a modified version of the McCulloch-Pitts neuron using an agent-based modeling approach. These neurons interact with surrounding neurons in the same way as MP neurons, i.e. by sending and receiving stimuli consisting of positive or negative integers, but their output is calculated by a simple agent-based representation of receptors and ion channels rather than by input summation. This representation also allows us to include phenomena such as absolute and relative refractory periods and the repolarization of a neuron that has received stimuli but not fired. We have used these neurons to implement a simulation of neuronal activity in the rat hippocampus using Repast Simphony and Repast HPC, and demonstrated that this model produces a more realistic simulation of hippocampal activity than a similar model using standard MP neurons. This multi-scale model of brain activity will allow us to perform additional studies on how changes to neuronal behavior representing injury or therapeutic enhancement may affect network activity at the global level.

